# Genome replication dynamics of a bacteriophage and its satellite reveal strategies for parasitism and viral restriction

**DOI:** 10.1093/nar/gkz1005

**Published:** 2019-10-31

**Authors:** Zachary K Barth, Tania V Silvas, Angus Angermeyer, Kimberley D Seed

**Affiliations:** 1 Department of Plant and Microbial Biology, University of California, Berkeley, Berkeley, CA 94720, USA; 2 Chan Zuckerberg Biohub, San Francisco, CA 94158, USA

## Abstract

Phage-inducible chromosomal island-like elements (PLEs) are bacteriophage satellites found in *Vibrio cholerae*. PLEs parasitize the lytic phage ICP1, excising from the bacterial chromosome, replicating, and mobilizing to new host cells following cell lysis. PLEs protect their host cell populations by completely restricting the production of ICP1 progeny. Previously, it was found that ICP1 replication was reduced during PLE(+) infection. Despite robust replication of the PLE genome, relatively few transducing units are produced. We investigated if PLE DNA replication itself is antagonistic to ICP1 replication. Here we identify key constituents of PLE replication and assess their role in interference of ICP1. PLE encodes a RepA_N initiation factor that is sufficient to drive replication from the PLE origin of replication during ICP1 infection. In contrast to previously characterized bacteriophage satellites, expression of the PLE initiation factor was not sufficient for PLE replication in the absence of phage. Replication of PLE was necessary for interference of ICP1 DNA replication, but replication of a minimalized PLE replicon was not sufficient for ICP1 DNA replication interference. Despite restoration of ICP1 DNA replication, non-replicating PLE remained broadly inhibitory against ICP1. These results suggest that PLE DNA replication is one of multiple mechanisms contributing to ICP1 restriction.

## INTRODUCTION

Viral satellites are found in all domains of life and can have a profound impact on their helper viruses and their host cells (1–3). These sub-viral agents are known to worsen disease in humans (4) as well as plants (5), provide bacterial pathogens with toxins necessary for virulence (6), and serve as anti-viral immune systems in both single celled eukaryotes (7) and bacteria (8). As the parasites of viruses, satellites face distinct challenges in their life cycles. Viruses typically need to subvert host cell nucleic acid metabolism in order to replicate their genome. In turn, viral satellites must find a way to subvert the subverters, so that the satellite's genome can be replicated and mobilized in addition to, or at the exclusion of, the helper virus.

Within bacteria, four phylogenetically unrelated families of tailed-bacteriophage satellites have been discovered. These include satellite phage P4 and its relatives found in *Escherichia coli* (9,10), the phage inducible chromosomal islands (PICIs) widespread throughout Firmicutes (11), the PICI-like elements (PLEs) found in epidemic isolates of *V. cholerae* (12), and the recently discovered Gram-negative PICIs found in Enterobacteriales and Pasturellales (13). Certain details of the life cycles of PLEs and their helper phage, ICP1, distinguish PLEs from other bacteriophage satellites. Both P4 and the well characterized subfamily of PICIs referred to as staphylococcal pathogenicity islands (SaPIs) confer partial restriction of their helper phages (14,15). In contrast, PLEs completely restrict ICP1 production when they are able to progress through their replication cycle (12). This allows PLEs to function as effective abortive infection systems: individual ICP1 infected cells die, but since no phage are produced, the population as a whole is protected (12). PLEs’ more severe restriction of its helper phage likely relates to ICP1’s life cycle. ICP1 is only known to produce lytic infections that kill the host cell (16). In contrast, both P4 and PICIs parasitize temperate phages which occasionally integrate into the genomes of the cells they infect. For P4 and PICIs, it is not uncommon to find a helper phage and its satellite lysogenizing the same strain (9,17). Since satellites rely on their helpers for mobilization, there can be intrinsic benefits to a low level of helper phage production that allows for co-lysogeny. If ICP1 kills every cell it can infect, cells that are potential hosts for PLEs, then it is to the PLEs’ benefit to completely restrict the production of infectious ICP1 progeny.

PLEs’ use of ICP1 as a helper virus also has implications for PLEs’ genome replication strategy. P4’s helper phage is known to rely on host-encoded machinery (18), and while the replication of PICI helper phages has not been extensively characterized, comparative genomics suggest that some of the characterized PICI helpers hijack host cell replication machinery (19,20). Similar to their helpers, both P4 and PICIs must redirect cellular machinery to the satellite genomes (21–23). The better characterized PICIs (i.e. SaPIs) have replication initiators that possess helicase activity and primases. P4 makes use of the same activities for its own replication initiation, but the initiating helicase and the primase are fused into a single protein. When these replication genes are expressed they are sufficient to drive autonomous satellite replication within the host cell (21,23) Like many well-studied lytic phages, ICP1 differs from the helper phages exploited by P4 and PICIs by encoding its own replication machinery (16). PLEs must therefore use a separate DNA polymerase from ICP1, or hijack ICP1’s DNA polymerase for their own replication. Either possibility provides a novel twist to bacteriophage satellite DNA replication.

For its own replication, ICP1 encodes a Pol-I type DNA polymerase and a helicase-primase with a Gp4d helicase domain like the *E. coli* phage T7 (24–26). The T7 replisome is one of the best characterized replisomes and is simpler in its components than most other replication complexes. Only four proteins are needed to reconstitute T7’s replisome *in vitro* (25): DNA polymerase; host-encoded thioredoxin, which acts as a processivity factor for the polymerase; helicase-primase, which in addition to possessing both helicase and primase activity has single stranded DNA binding activity and loads the DNA polymerase; and single stranded DNA binding protein that aids in replisome assembly and is necessary for lagging strand synthesis. The relative simplicity of the ICP1 replisome may make it an attractive target for exploitation by PLE. Indeed, PLE replication through use of ICP1’s replisome would then be in line with PLE’s reliance on ICP1 for multiple steps in the PLE life cycle. To excise PLE from the bacterial chromosome, the PLE integrase requires an ICP1-encoded recombination directionality factor (27), and PLE also requires the same viral receptor as ICP1 for transduction (12), suggesting that PLE is packaged into ICP1 capsids just as P4 and PICIs are packaged into the capsids of their helper phage (2).

PLE’s severe parasitism of ICP1 has necessitated ICP1’s evolution of counter defenses. ICP1 host range on different PLEs varies among ICP1 isolates in a manner reminiscent of host-parasite co-evolution (12). So far, five distinct PLEs have been identified, and there has been a temporal succession of these elements, with a new PLE emerging around the same time as the previous PLE disappears from sequenced isolates. PLEs are prevalent, occurring in ∼25% of *V. cholerae* isolates spanning a 60-year collection period (12). PLE(+) *V. cholerae* have been isolated from cholera patient stool samples alongside ICP1, suggesting that ICP1 infection, and PLE parasitism of ICP1, takes place within human hosts (8,12,27,28). ICP1 isolates appear to have multiple strategies to overcome PLE (12), but the only mechanism identified so far is a phage-encoded CRISPR-Cas system (8,28). Among PLE genes, only the PLE integrase has a recognized function (27), and the precise mechanism(s) by which PLEs restrict ICP1 continue to elude.

**Figure 1.**
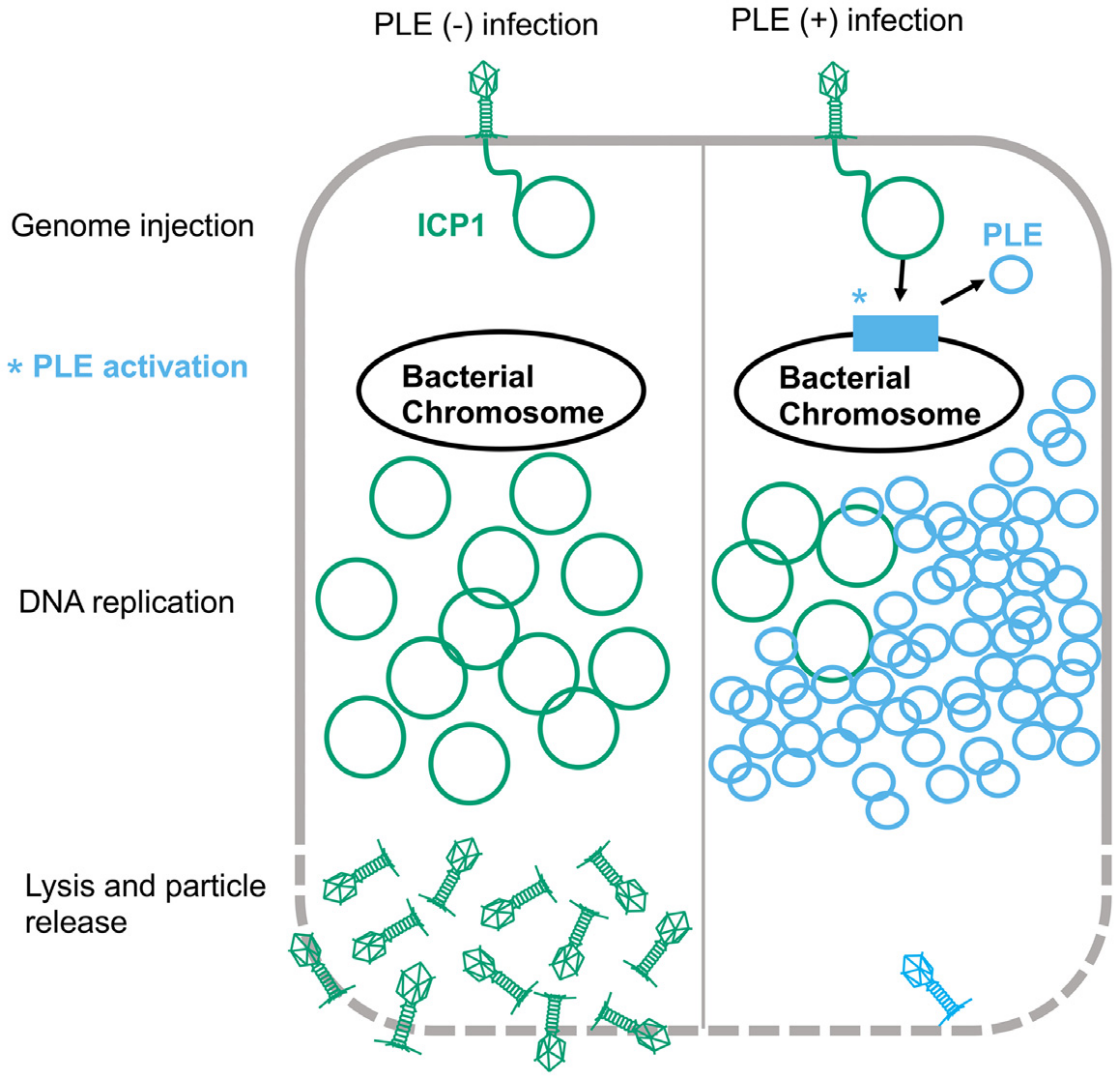
Model of ICP1 infection in PLE(−) and PLE(+) *V. cholerae*. ICP1 injects its DNA into *V. cholerae*; prior to DNA replication, ICP1 activity leads to PLE activation and excision. ICP1 DNA replication is reduced in the PLE(+) cell where the PLE replicates to high copy. Finally, the cell lyses and releases infectious particles. No ICP1 particles and a low number of PLE transducing particles are released from the PLE(+) cell.

Given the crucial role of genome replication in viral propagation, the interface of PLE and ICP1 DNA replication is likely tied to PLEs’ ability to restrict ICP1. Previous work showed that PLEs can replicate upwards of 1000-fold following ICP1 infection (12) (Figure 1). This increase in PLE copy is accompanied by a 3 to 4-fold inhibition of ICP1 DNA replication. Curiously, PLEs do not transduce well under laboratory conditions, producing fewer than one PLE transducing unit per infected cell. Further, in these laboratory conditions, four of the five PLEs integrate seemingly randomly into one of *V. cholerae's* many *V. cholerae-*repeats (VCRs), but for PLE(+) *V. cholerae* isolates from nature, each of the four PLEs always occupies the same VCR, indicating that horizontal transmission may be rare (12). This suggests that transduction may play a minor role in the PLE life cycle, and/or that it may be infrequent. The discrepancy between robust PLE replication and poor PLE mobilization led us to investigate the requirements for PLE replication, and whether PLE may bolster its anti-phage activity through increasing its copy number.

Here, we define the replicon of PLE 1 (hereafter referred to as PLE), identifying an origin of replication and a PLE-encoded replication initiation factor. The PLE replication initiator belongs to the RepA_N family of proteins, and to our knowledge is the first RepA_N protein functionally characterized in a Gram-negative bacterium. While PLE replication is not necessary to provide anti-phage immunity against ICP1, loss of PLE replication does restore the level of ICP1 DNA replication, and allows for a low level of ICP1 virion production, suggesting that PLE replication may be one of perhaps several parallel mechanisms that work in tandem to restrict ICP1.

## MATERIALS AND METHODS

### Strains and culture conditions

*V. cholerae* strains used in this study are derived from E7946. Bacteria were routinely grown on LB agar plates and in LB broth with aeration at 37°C. Antibiotics were supplemented as appropriate at the following concentrations: 75 μg/ml kanamycin, 100 μg/ml spectinomycin, 1.25 or 2.5 μg/ml chloramphenicol (*V. cholerae* for broth or plate conditions, respectively), 25 μg/ml chloramphenicol (*E. coli*), 100 μg/ml streptomycin. A detailed list of all strains used throughout this study can be found in Supplementary Table S1. ICP1_2006_E engineered to lack CRISPR-Cas (ΔCRISPR,*Δcas2-3*) (27) was used for all experiments. Phage titers were determined using a soft agar overlay method wherein ICP1 was allowed to adsorb to *V. cholerae* for 10 min at room temperature before the mixture was added to molten LB soft Agar (0.3%) and poured onto 100 mm × 15 mm LB Agar plates. Plaques were counted after overnight incubation at 37°C. Efficiency of plaquing on mutant PLE strains was determined by dividing the phage titer obtained on the mutant PLE(+) strain by the phage titer obtained on PLE(−) strain.

### Generation of mutant strains and constructs

*V. cholerae* mutants were generated through natural transformation or *sacB* counter selection. Natural transformation was performed as described previously (29). For gene knockouts, splicing by overlap extension (SOE) PCR was used to generate deletion constructs with a spectinomycin resistance cassette flanked by frt recombination sites. Following selection of spectinomycin resistant mutants, a plasmid bearing an IPTG inducible Flp recombinase was mated into transformants and Flp expression was induced to generate in-frame deletions. The plasmid was cured by growing mutants under inducing conditions with 300μg/ml streptomycin. For plasmid expression constructs, a derivative of the pMMB67EH vector with a theophylline controlled riboswitch was used as previously described (27). For strains made via *sacB* counter selection, a marker-less deletion construct was generated using SOE PCR, and cloned into a pCVD442 suicide vector bearing the *sacB* counter selectable marker and an ampicillin resistance marker via Gibson assembly. LB Agar 10% sucrose plates were used to select for *sacB* loss and recombination of the mutant allele. All constructs were confirmed with DNA sequencing over the region of interest and primer sequences are available upon request.

### Real-time quantitative PCR

qPCR experiments were performed as previously described (12). Briefly, liquid cultures were infected with ICP1 at a Multiplicity of Infection (MOI) of 2.5 at OD_600_ = 0.3. Samples were taken at 0 and 20 min post-infection, and boiled before serving as templates for IQ SYBR qPCR reactions. For assays involving induction of *repA*, 2 ml cultures were grown with 1.25 μg/ml chloramphenicol for plasmid maintenance and induced for 20 min prior to infection using a final concentration of 1.5 mM theophylline and 1 mM IPTG starting at OD_600_ = 0.17. Primers used for qPCR are listed in Supplementary Table S2.

### Nanoluciferase reporter assay

Liquid cultures were grown to an OD_600_ = 0.3. Immediately prior to infection and at 4 min intervals following infection, 100 μl of culture infected at an MOI of 2.5 was added to an equal volume of cold methanol. Nanoluciferase production was measured using the Nano-Glo^®^ Luciferase Assay System (Promega). The NanoGlo substrate was diluted 50-fold in the NanoGlo buffer and for each sample, 50 μl of sample and 50 μl of reaction mix were added per well in a black 96 half-well plate. Luminescence was read over 7 min at room temperature with 10 s shaking between reads. For each biological replicate, the average of 10 reads was used.

### Protein purification

*E. coli* BL21 cells containing a His_6_-SUMO fusion to *repA* were grown to OD_600_ = 0.5 at 37°C and induced with IPTG to a final concentration of 0.5 mM. The cultures were then shifted to 16°C and grown for 24 h. Cells were centrifuged and resuspended in lysis buffer (50 mM Tris–HCl pH 8, 200 mM NaCl, 1 mM BME, 0.5% Triton-X, 50 mM imidazole, 1 Pierce™ Protease Inhibitor Mini Tablet (Thermo Scientific) and sonicated. Cell debris was removed by centrifugation (29 097 × g for 40 min), and the lysate was applied to a nickel resin affinity column (HisPur Ni-NTA Resin, Thermo Scientific). The column was washed with two column volumes of wash buffer (50 mM Tris–HCl pH 8, 200 mM NaCl, 1 mM BME, 50 mM imidazole), one column volume of an additional high salt wash (50 mM Tris–HCl pH 8, 2 M NaCl, 1 mM BME, 50 mM imidazole) to remove any residual DNA, and then eluted with elution buffer (50 mM Tris–HCl pH 8, 200 mM NaCl, 1 mM BME, 300 mM imidazole). The eluate was dialyzed with sizing buffer (50 mM Tris–HCl pH 7.5, 150 mM NaCl, 1 mM Dithiothreitol) in 10K MWCO SnakeSkin dialysis tubing (Thermo Scientific), and the His_6_-Sumo-tag was cleaved with 1 μl SUMO protease per 100 μg of protein. The eluate was fractionated on a HiLoad 16/60 Superdex 75 size-exclusion column (GE Healthcare) and fractions were analyzed using SDS page. Protein was concentrated using an Amicon Ultra 15 ml 3K NMWL centrifugal filter (Millipore Sigma).

### Fluorescent labeling of oligos

Single stranded oligos were labeled with 5′-TAMRA using the 5′ EndTag™ Nucleic Acid Labeling System (Vector Laboratories) following the manufacturer's protocol, 0.6 nM of single stranded probe was labeled with a 5 mg/ml solution of tetramethylrhodamine-5-maleimide in DMSO. Following end labeling, the labeled single stranded probe was annealed to its complementary sequence by mixing equimolar concentrations of complementary oligos in water, heating to 65°C for 4 min, and then allowing the reaction to return to room temperature. Probe sequence is available in Supplementary Table S2.

### Electrophoretic mobility shift assays (EMSAs)

80 nM probe was incubated with purified RepA at 30°C for 20 min in 20 μl reactions with 10 mM HEPES pH 7.8, 10% glycerol, 1 μM TCEP, 10 mM MgCl_2_ and 0.4 μg poly(dI-dC) (Sigma-Aldrich) serving as a nonspecific competitor. The full reaction volume was then loaded onto 8% acrylamide 0.5× Tris-borate gels and ran for 20 min at 120 V before visualization.

### Preparation of phage infection samples for DNA sequencing

A 6 ml bacterial culture was infected at OD_600_ = 0.3 with ICP1 at an MOI of 1. At the indicated time points, 1 ml was removed from the culture tube and mixed with 1 ml ice cold methanol to stop DNA synthesis. These samples were pelleted at 21 694 × g for 2 min at 4°C. The methanol was removed through aspiration and the pellet was washed with 1 ml cold phosphate-buffered saline. Pellets were frozen in liquid nitrogen and stored at −80°C until total DNA was isolated using the QIAGEN DNeasy Blood and Tissue Kit. Sequencing libraries were prepared using NEBNext^®^ Ultra™ II FS DNA Library Prep Kit. Paired-end sequencing (2 × 150 bp) was performed on an Illumina HiSeq4000 (University of California, Berkeley QB3 Core Facility).

### DNA-seq reads mapping

Illumina sequencing reads for each timepoint were mapped to the appropriate reference sequence using Bowtie 2 v2.3.4.1 (30) with default settings except for the following: ‘–end-to-end’ and ‘–very-sensitive’. Mapping files were sorted and indexed with samtools v1.5 (31) and binned with breseq BAM2COV v0.33.0 (32): ICP1 1000bp, PLE 150bp. Read coverage was normalized by the total number of reads that mapped to the reference. Triplicate experiments were then averaged and plotted with the matplotlib module v3.0.3 in Python (33). GC skew was calculated over a 1000 bp sliding window. For plotting the abundance of a specific genome in a sample, the genome per million (GPM) was calculated in the same manner as the previously described transcripts per million (34).

### Protein structure visualization

Protein structure figures were generated using PyMOL (The PyMOL Molecular Graphics System, Version 2.0 Schrödinger, LLC). Protein structure alignments were generated using the cealign command (35). The electrostatic distribution was determined and visualized using the PDB2PQR server, and the APBS plugin for PyMOL (36).

### Transduction assays

Transduction assays were performed as previously described (12). Briefly, donors were grown to OD_600_ = 0.3 and infected with ICP1 at an MOI of 5. Cultures were incubated for 5 min before being washed with fresh LB to remove unbound phage. The infected cultures were incubated for 30 min, and 100 μl lysate was added to 100 μl of an overnight culture of the recipient strain. The mixture was incubated for 1 h at 37°C with aeration before plating on selective media.

### Efficiency of center of infection assay

Cultures were grown to an OD_600_ = 0.3, at which time they were infected at an MOI of 0.1 and incubated for 7.5 min to allow phage attachment before being diluted 2500-fold in warm LB. 500 μl of this dilution was collected and treated with 20μl chloroform to enumerate phage input. The diluted cultures were diluted further, 10 and 100-fold into two additional tubes containing 2 ml LB. The dilution series of infected cells was then returned to the incubator. Infected cells were collected at 35 min post initial infection. A plaque assay was performed by adding the infected cells to PLE(−) cells to measure the center of infection for the strains of interest.

## RESULTS

### PLE alters and diminishes ICP1 replication

**Figure 2.**
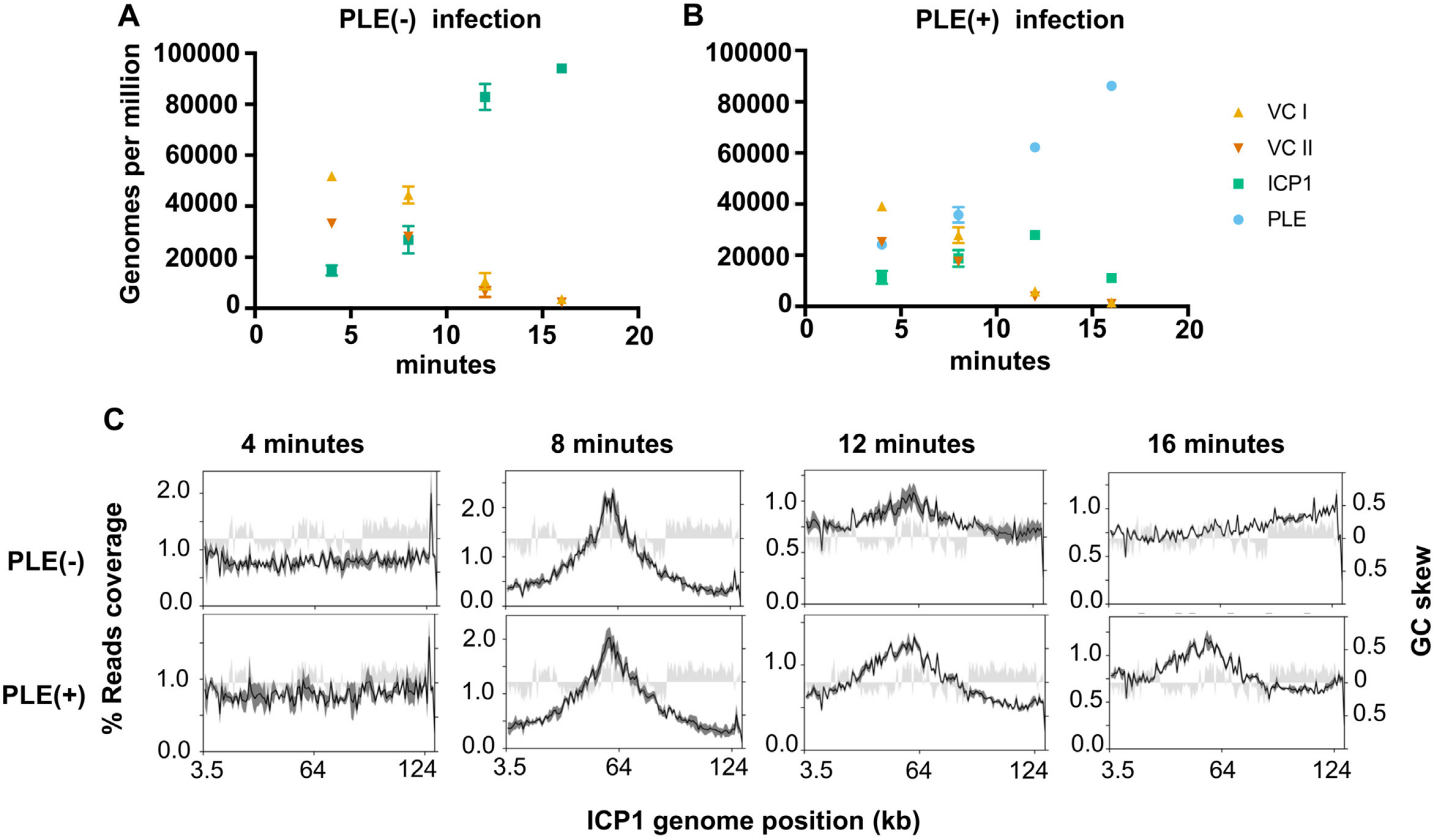
PLE robustly replicates following infection while altering ICP1 replication. (A and B) Genomes per million (GPM) of total DNA mapping to the *V. cholerae* large (VC I) and small (VC II) chromosomes, ICP1, and the PLE across an infection time course in PLE(−) (**A**) and PLE(+) (**B**) *V. cholerae*. Samples were taken at 4, 8, 12 and 16 min post-infection, data show the average and standard deviation of three independent experiments. (**C**) Percent reads coverage plots across the ICP1 genome during PLE(−) (top) and PLE(+) (bottom) infection. For each time point, the percent reads coverage across the genome for three biological replicates was determined. The average percent reads coverage is shown as a black line, while standard deviation appears as dark gray shading around the line. The GC skew (right axis) is shown as light gray shading.

PLE was previously shown to replicate to high copy during ICP1 infection and reduce ICP1 DNA replication compared to a PLE(−) control (12) (Figure 1), however, these results were obtained through qPCR and only assessed a single ∼100bp target sequence. To obtain a more complete understanding of PLE replication dynamics and the PLE’s impact on ICP1 replication kinetics, we performed deep sequencing of total DNA during an ICP1 infection time course using PLE(−) and PLE(+) *V. cholerae*. ICP1 produces new progeny virions by 20 min post-infection in PLE(−) cultures, and PLE(+) cultures lyse 20 min post-infection (12), therefore to evaluate total DNA content in infected cells at early, middle and late time points (while avoiding potential DNA loss due to lysis), we collected samples at 4, 8, 12 and 16 min post-infection. Total DNA from samples at each time point was sequenced on an Illumina HiSeq and the resulting sequencing reads were mapped against the *V. cholerae*, ICP1, and PLE genomes. Consistent with the anticipated rapid kinetics of ICP1 infection in PLE(−) *V. cholerae*, the abundance of ICP1 reads increased within 8 min post-infection and ICP1 DNA comprised roughly half of the total DNA content by 16 min post-infection (Supplementary Table S3). To account for the relatively small size of the ICP1 genome compared to the *V. cholerae* chromosomes, we normalized the reads mapped per element to element length and the total reads per sample to determine the genomes per million (GPM) of each entity in the samples. Prior to infection, the GPM for the *V. cholerae* large chromosome is higher than that for the small chromosome (Figure 2A), consistent with previous studies showing that replication of the small chromosome initiates after the large chromosome, leading to roughly synchronous termination (37), and that replication initiation of the small chromosome requires duplication of certain loci in the large chromosome (38). Following infection, ICP1 DNA replication robustly overtakes the cell and phage genomes are more abundant than copies of the *V. cholerae* large and small chromosomes by 12 min post-infection (Figure 2A). In contrast, ICP1 DNA replication is less robust in the presence of PLE. Specifically, the proportional abundance of ICP1 DNA is relatively unchanged at 4 and 8 min post-infection of PLE(+) cells, but ICP1 DNA replication begins to dramatically lag by 12 min post-infection compared to PLE(−) infection (Supplementary Table S3). In the PLE(−) condition, ICP1 relative reads abundance doubles from roughly one quarter to one half of total reads between 12 and 16 min post-infection, while in the PLE(+) condition ICP1 abundance increases very little between 12 and 16 min post-infection (Supplementary Table S3). The defect observed in ICP1 replication correlates with PLE’s own robust replication. By 8 min post-infection, PLE is already the most abundant element in terms of copy number (Figure 2B). Between 8 and 16 min post-infection, the abundance of PLE DNA grows to comprise ∼19% of total reads, overtaking ICP1 in total DNA (Supplementary Table S3). In terms of genome copy at 16 min post-infection, PLE outnumbers ICP1 ∼8-fold (Figure 2B). The temporal dynamics of PLE and ICP1 DNA replication support the notion that interference of ICP1 replication may be linked to the PLE’s own replication.

In addition to monitoring the relative changes in abundance of discrete genetic elements during phage infection, we evaluated the profiles of sequence coverage across ICP1 and PLE genomes (Figure 2C, Supplementary Figure S1). While the distribution of reads across the ICP1 genome was similar in PLE(+) and PLE(−) conditions at 8 min post-infection, ICP1’s coverage profile was markedly different at 12 and 16 min post-infection between the two conditions (Figure 2C). At 8 min post-infection, a peak in ICP1 reads can be seen near the 60 kb position, and reads abundance decreases with increasing distance from that point. The observed pattern in ICP1, which is present in both PLE(+) and PLE(−) infections, is consistent with the predicted coverage of an element that replicates bidirectionally through theta-replication from a single origin of replication (ori) (39). At 16 min post-infection in the PLE(−) condition the peak reads abundance shifts to one end of ICP1’s genome (Figure 2C). These results suggest activation of an additional ICP1 ori late in infection. Additionally, the distribution of reads decreases gradually in the upstream direction from the peak, and sharply drops downstream of the peak. Such a distribution is suggestive of a rolling circle mode of replication (39), which is consistent with a number of phages that are known to transition to rolling circle late in infection (26). By contrast, at 16 min post-infection in PLE(+) *V. cholerae*, the profile of ICP1 reads more strongly resembled the profile at 8 min post-infection than it did to the coverage profile 16 min post-infection in the PLE(−) condition. The change in ICP1 reads distribution suggests that PLE might alter ICP1 replication origin choice and impair the progression from theta to rolling circle replication.

Intriguingly, the reads peak at near the end of ICP1’s annotated genome was prominently visible at 4 min post-infection, before ICP1 replication has taken place (Figure 2C). We speculated that this reads peak corresponds with the terminus of infecting ICP1 particles prior to genome circularization as it has previously been established that termini can lead to sequencing biases following DNA library preparation (40). We found that this reads bias was also present in DNA from purified phage particles (Supplementary Figure S2). PhageTerm, which identifies phage termini and packaging methods (40), identified this reads peak as a packaging (pac) site and predicted a headful packaging mechanism for ICP1. The terminus is located in a 1.3 kb orfless space between *gp1* and *gp2* (Supplementary Figure S2A and B). PhageTerm predicts the location of the pac site at 431 bp on the annotated (+) strand, and 891 bp on the annotated (−) strand (Supplementary Figure S2C). The loss of this peak by 8 min post-infection (Figure 2C) likely reflects circularization of the phage genome following cell entry. Together, ICP1’s changing coverage profile over the course of infection, and PhageTerm analysis of phage particle DNA suggests that rolling circle initiation and genome packaging may be linked for ICP1. That the shift in ICP1’s coverage profile was profoundly reduced in the PLE(+) background suggests that PLE interferes with the rolling circle mode of ICP1 replication, potentially preventing the switch from theta replication. This interference of rolling circle replication may perturb later steps (i.e. DNA packaging) necessary for ICP1 to complete its life cycle.

### PLE encodes its own replication initiator, but does not replicate autonomously

**Figure 3.**
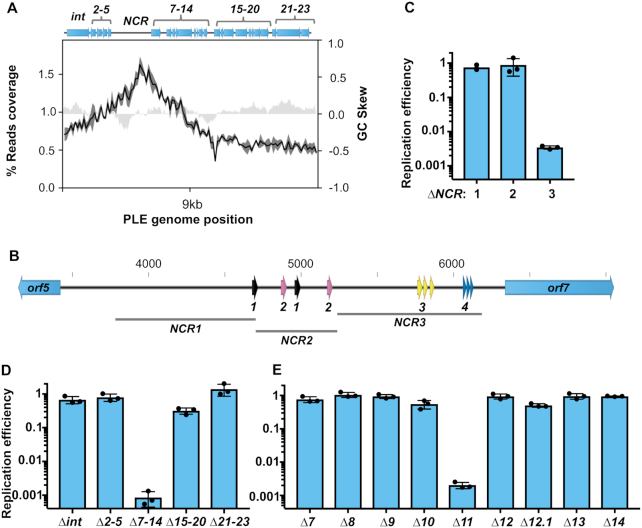
A single PLE-encoded ORF and a noncoding region are necessary for PLE replication. (**A**) A representation of the PLE genome (top) with average reads coverage of PLE 8 min post ICP1 infection plotted below. The percent reads coverage was determined for three biological replicates and is shown as a black line, while standard deviation appears as dark gray shading around the line. The GC skew (right axis) is plotted as light gray shading. Gene clusters mutated for analysis are labelled. (**B**) PLE’s noncoding region (NCR) between *orf5* and *orf7*, with repeat sequences shown as arrows. Repeat sequences share colors for each repeat type, and are designated as repeats 1, 2, 3 or 4. Regions of the NCR deleted for analysis in (C) are shown. Panels C–E: replication of PLE mutants 20 min post-infection with ICP1 as assessed by qPCR. Replication efficiency is relative to a wild-type PLE control. (**C**) Replication of ΔNCR mutants. (**D**) Replication of PLE gene cluster knockouts. (**E**) Replication of individual gene knockouts of the ORFs contained in cluster *7–14*.

To better understand the relationship between PLE and ICP1 DNA replication, we next sought to identify the constituents of the PLE replicon. The PLE genome is 18kb and organized into multiple predicted gene clusters (Figure 3A). Between PLE *orf5* and *orf7*, is a 2.7 kb non-coding region (NCR) which has four repeat sequences (Figure 3B, Supplementary Figure S3). Frequently, repetitive sequences serve as binding sites for replication machinery at phage and plasmid origins of replication (41). Within bacterial genomes, there is also a bias for coding sequence in the leading strand (42), and this is consistent with an ori being between divergently transcribed operons. These features led us to hypothesize that the PLE NCR serves a function in replication. This was further evidenced by PLE’s replication reads profile, which showed a peak approximately 1kb upstream of *orf7* at 8 min post-infection, suggesting that the PLE ori is located in the NCR (Figure 3A). To test if the NCR contained sequence necessary for PLE replication, we generated three strains designated NCR1, NCR2 and NCR3 that each possessed a 0.5–1kb deletion within the NCR excluding predicted promoters for *orf5* and *orf7* (Figure 3B). Following ICP1 infection we found that NCR1 and NCR2 were dispensable for PLE replication, however, NCR3, containing repeat sequences 3 and 4, was necessary for replication (Figure 3C). This suggested that the PLE ori was contained within NCR3, and that repeat 3 and/or repeat 4 may be involved in replisome recruitment. We next sought to determine whether any predicted PLE open reading frames (ORFs) are necessary for PLE replication. We began by screening PLE gene cluster knockouts (27) and we observed that one gene cluster, containing *orf7* through *orf14*, was necessary for PLE replication (Figure 3D). To identify the ORF responsible, we constructed individual gene knockouts within this cluster and screened for replication defects during ICP1 infection. We found that a single open reading frame, *orf11* (https://www.ncbi.nlm.nih.gov/protein/AGG09405.1/), was necessary for PLE replication (Figure 3E). Given the requirement of *orf11* for PLE replication, and further analyses supporting its designation as a replication initiation protein (discussed below) we designated *orf11* as *repA*.

We next wanted to check if ICP1 induces expression of *repA*. We tested this by infecting a *ΔrepA* reporter strain with nanoluciferase under the native *repA* promoter. Samples from infected cultures and uninfected controls were taken just prior to infection and at 4 min intervals following infection. The luminescence activity of the infected strain was noticeably higher at 8 min-post infection, and continued to climb at 12 and 16 min, confirming that ICP1 infection activates expression of *repA* (Supplementary Figure S4).

**Figure 4.**
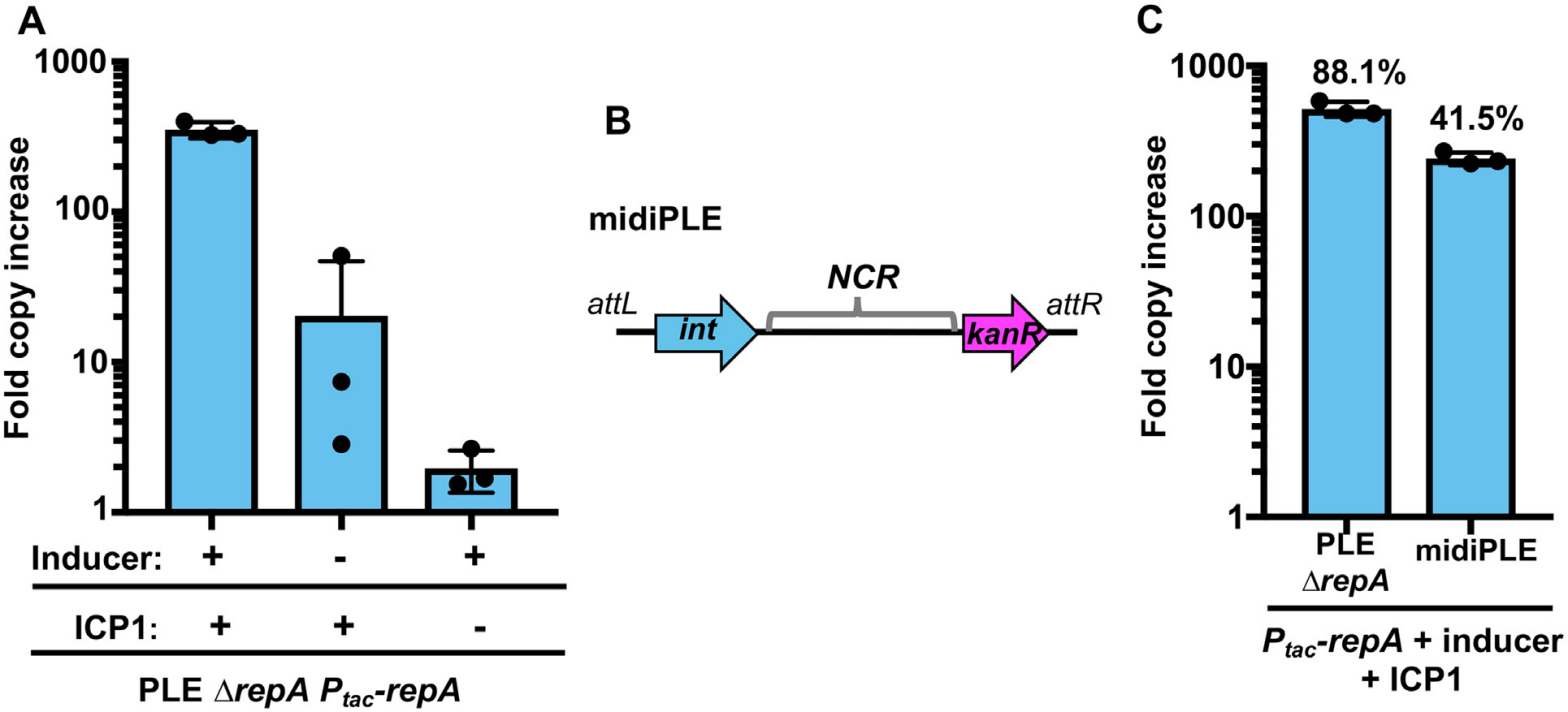
RepA drives PLE replication in the presence of ICP1. (**A**) RepA complementation of PLE *ΔrepA* as assessed by qPCR. PLE fold copy increase 20 min post-infection is shown in different combinations with ICP1 and the inducer of the complementation construct. (**B**) A diagram of the midiPLE construct used to assess the minimal requirements for PLE replication (not to scale). Attachment sites, the PLE integrase, and the noncoding region (NCR) are present along with a kanamycin resistance gene (*kanR*). (**C**) Replication of a RepA complemented *ΔrepA* strain and midiPLE 20 min post ICP1 infection. The replication of these strains was compared to a wild-type PLE control, and the relative replication is displayed as a percentage above the bars.

In both P4 and SaPIs, satellite replication is autonomous following the satellite's transcriptional activation by the helper phage (21,43). Having determined that PLE-encoded *repA* is induced upon ICP1 infection and necessary for PLE replication, we sought to elucidate if expression of *repA* was sufficient to drive autonomous replication of PLE. We complemented PLE *ΔrepA* with ectopically expressed *repA* and measured PLE copy number increase in the presence and absence of phage. RepA expression was able to drive PLE replication, but only in cells infected by ICP1 (Figure 4A). Our infected uninduced culture exhibited a low level of PLE replication, presumably due to leakiness of the expression construct. Consistent with this, ICP1 was unable to drive replication of the *ΔrepA* PLE complemented with an induced empty vector control (Supplementary Figure S5). This result shows that RepA is necessary for PLE replication.

To rule out the possibility that PLE replication requires additional PLE genes activated by ICP1 that may have been missed in our genetic screen due to redundancy, we next set out to define the minimal unit required for PLE replication. Previous work showed that ICP1 infection triggers excision of a ‘miniPLE’, consisting of the PLE-encoded integrase together with a kanamycin resistance marker flanked by the PLE attachment (att)-sites (27). We built on this existing platform and constructed a ‘midiPLE’ which differs only by the presence of the NCR containing the PLE ori on the self-excising miniPLE (Figure 4B). The midiPLE replicated to a substantial level that was dependent on ICP1 infection and *repA* expression, replicating to about 40% the copy number of a wild-type control (Figure 4C). This result confirmed that *repA* and the PLE non-coding region are sufficient to drive PLE replication, but only during phage infection.

To determine if the capacity to drive PLE replication is unique to ICP1, we also tested PLE replication during infection by ICP3, an unrelated T7-like phage (16). Being a T7-like phage, ICP3 encodes a DNA polymerase and helicase-primase belonging to the same families as those of ICP1. To avoid the midiPLE being potentially degraded along with the host chromosome by ICP3, we complemented *ΔrepA* PLE with the ICP1-encoded recombination directionality factor PexA to stimulate PLE excision (27), as well as RepA. Due to shared promoters and the toxicity of inducing PexA, we were unable to induce expression of RepA prior to infection, precluding high levels of replication. Nevertheless, during infection by ICP1 under these conditions, the PLE replicated upward of 50-fold (Supplementary Figure S6). By contrast, the ICP3 infected cultures and uninfected controls did not show any evidence of PLE replication, suggesting that PLE replication may require components uniquely encoded by ICP1.

### The PLE-encoded replication protein RepA resembles Gram-positive plasmid initiation factors

**Figure 5.**
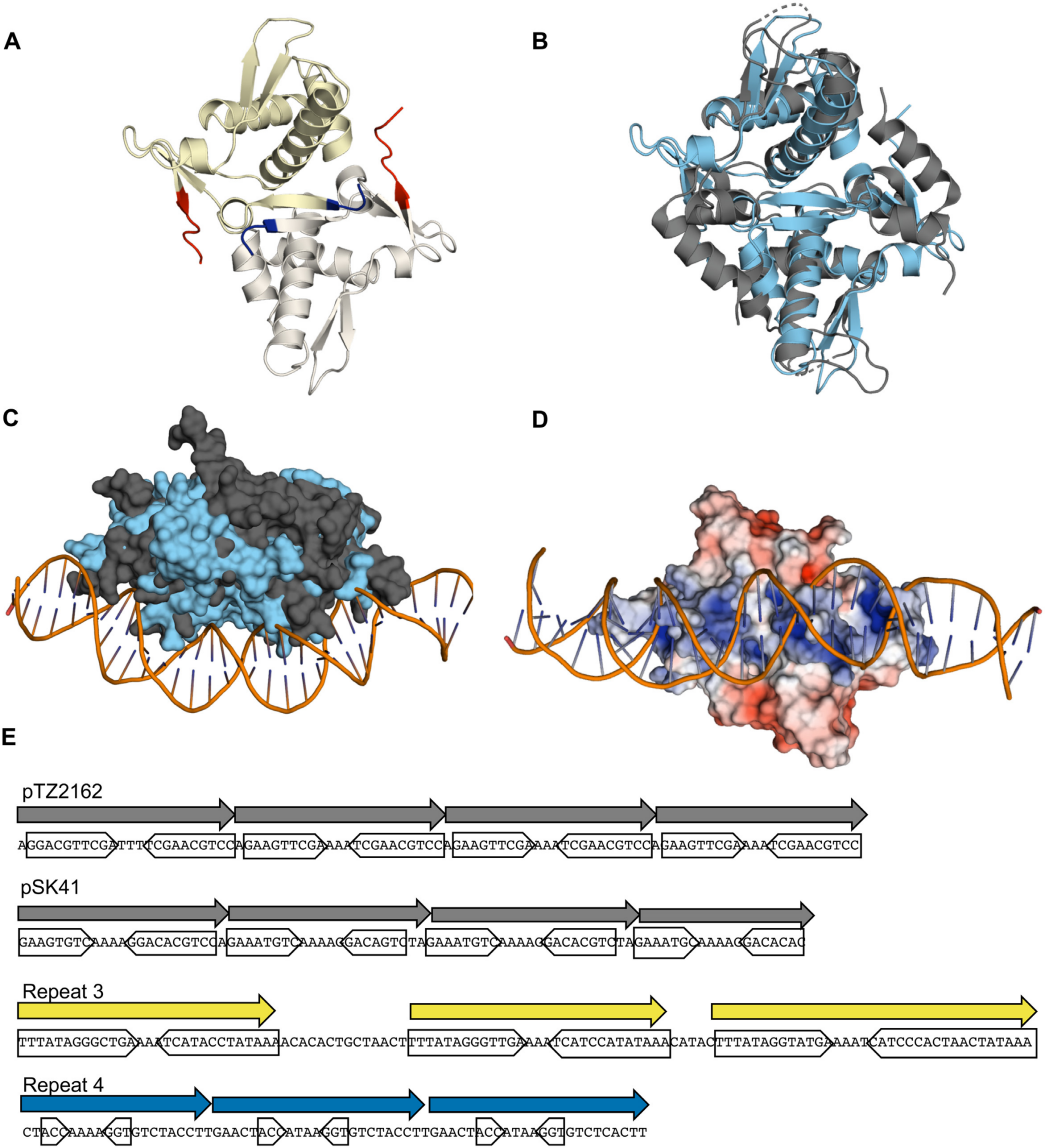
PLE RepA is a RepA_N family protein. (**A**) Cartoon representation of the PLE RepA-NTD dimer. Monomers are differently colored in yellow and white. The N and C termini of the monomers are colored blue and red respectively. (**B**) Alignment of the NTD dimers of PLE RepA and pTZ2162 RepA in light blue and dark grey, respectively, depicted in cartoon representations (RMSD = 4.527 over 176 residues). (**C**) Surface view of PLE RepA-NTD dimer in light blue aligned with pTZ2162 RepA-NTD dimer in dark gray bound to substrate DNA. (**D**) Electrostatic potential map, turned 90 degrees as (C), of PLE RepA-NTD dimer aligned to the pTZ2162 RepA-dsDNA bound structure. Positive (blue) and negative (red) charges are indicated on the surface. (**E**) Binding iterons for the RepA initiators of pTZ2162 and pSK41 are shown alongside repetitive sequences found in the putative PLE origin of replication. Direct repeats are denoted with an arrow, while the sequence comprising inverted sub-repeats is boxed. Sequence for the minus strand for PLE is shown to make the central poly-A tract apparent.

Although the structure and function of PLE’s RepA has not been previously elucidated, the X-ray crystal structure of the N-terminal domain (NTD) of RepA (RepA-NTD) has been solved and deposited in the Protein Data Bank (PDB ID: 4RO3) (Figure 5A). While primary sequence similarity (using BLASTP) is not evident, using Dali (44), we found that PLE RepA-NTD has substantial structural similarity to the pKS41 and pTZ6162 plasmid RepA proteins from *Staphylococcus aureus*, as well as more distant similarity to the replication protein DnaD from *Bacillus subtilis* (PDB ID: 4PTA, 4PT7, and 2v79). Both of the *S. aureus* RepA proteins serve as replication initiators for plasmids coding for multidrug resistance and belong to the RepA_N family of plasmid replication proteins. The RepA_N protein family is comprised mostly of initiation factors for theta-replication of plasmids found mainly in the Firmicutes (45,46). This protein family is named for the conservation of the NTD which structurally resembles the NTD of the Gram-positive primosome component DnaD (47). In the RepA_N family, the NTD mediates DNA binding, while the C-terminal domain (CTD) of these proteins appear to be specific to host genus, and may perform host specific functions (45). HHPRED (48) did not detect any substantial similarities to RepA’s CTD (expect >1 for all hits).

The PLE RepA-NTD structure aligns well with the crystal structures of the NTDs from *S. aureus* pTZ6162 and pSK41 RepA initiation factors, highlighting shared tertiary structure (Figure 5B, Supplementary Figure S7). Notably, all three proteins crystallized as dimers with monomers in the same orientation, suggesting a conserved dimer interface, and the potential for a conserved method for DNA binding. A crystal structure for the pTZ6162 RepA-NTD dimer bound to its cognate iteron dsDNA sequence has also been solved (PDB ID: 5kbj) (47), and we were able to align the PLE RepA-NTD to this structure (Figure 5C). In the original pTZ6162 structure, the surface of the protein that is bound to the DNA is electropositive (47), consistent with binding activity for the electronegative dsDNA sugar-phosphate backbone. The corresponding surface in the PLE RepA-NTD structure is electropositive as well, suggesting conserved DNA binding activity (Figure 5D). An electropositive DNA binding interface is also observed in the pSK41 RepA structure suggesting maintenance of a shared DNA binding region among these proteins (Supplementary Figure S8A–C). Notably, the DnaD-NTD is less electropositive along the corresponding surface, which is expected since the DnaD-NTD, despite its structural similarity to RepA_N-NTDs, does not bind DNA (Supplementary Figure S8D) (45). Given PLE RepA’s structural similarity to the RepA_N family, its similar electrostatic profile, and its shared role as a replication factor for a mobile genetic element, we conclude that PLE RepA belongs to the RepA_N protein family.

**Figure 6.**
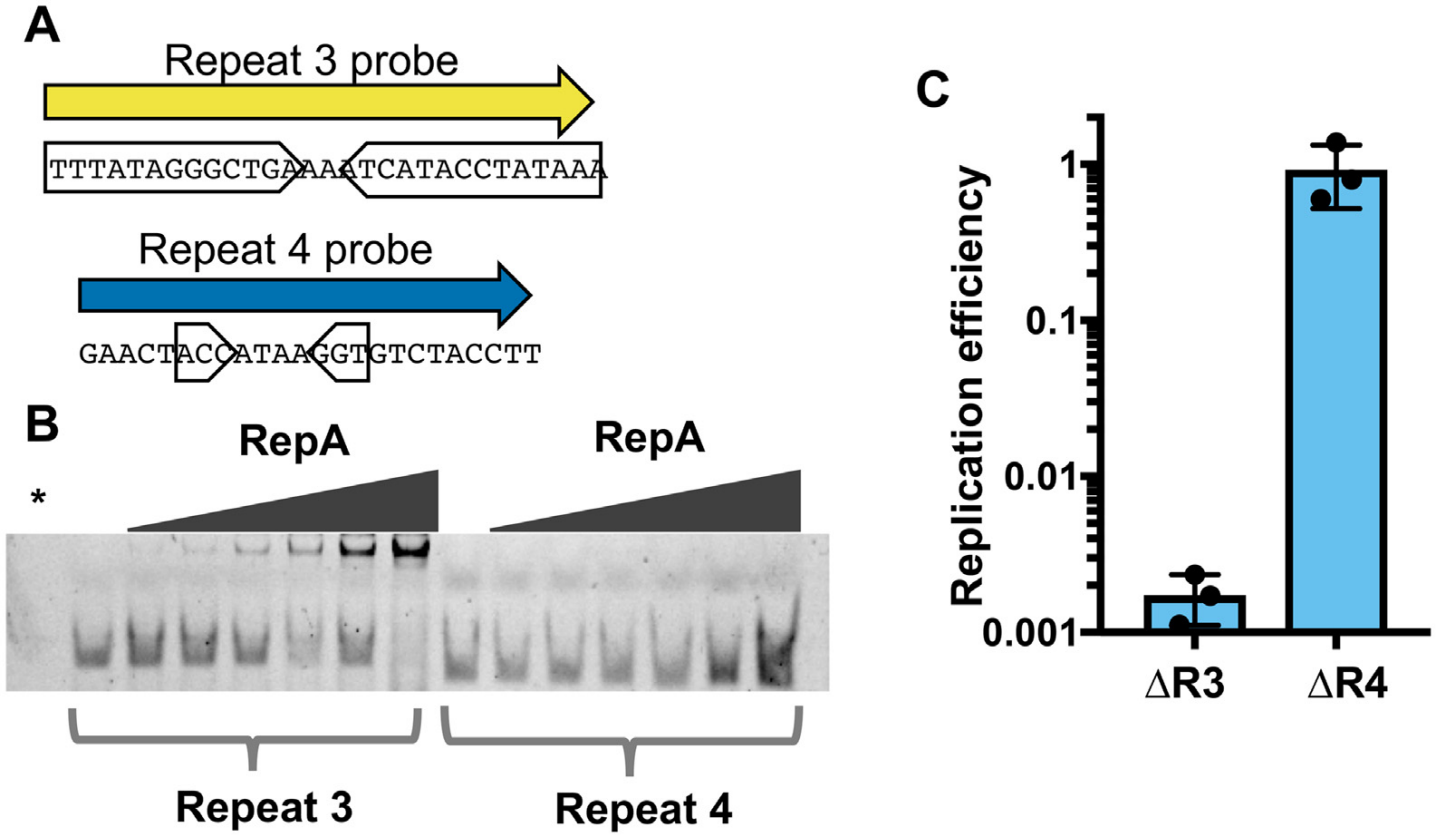
Repeat 3 serves as the PLE ori iteron sequence. (**A**) The nucleotide sequences of 5′ fluorescently labelled dsDNA probes used to test RepA specificity. Probe sequence was derived from the repetitive sequences found in PLE noncoding region 3 (NCR3). Inverted sub-repeats are boxed. (**B**) An electrophoretic mobility shift assay using the probes in (A). The * denotes a RepA(+) no DNA control. Additional replicates of this experiment are shown in Supplementary Figure S9. (**C**) The replication of PLE mutants with deletions spanning the repeat 3 (R3) and repeat 4 (R4) regions 20 min post-infection with ICP1 as assessed by qPCR. Replication efficiency is relative to a wild-type PLE control.

Like other replication initiation factors (41), RepA_N family proteins bind to repetitive iteron sequences at their cognate ori. Most characterized RepA_N iterons are semi-palindromic direct repeats, containing inverted repeats that converge on a poly-A tract (45) (Figure 5E). These same sequence features are apparent in repeat 3 and repeat 4 in NCR3 (Figure 5E), which is necessary for PLE replication (Figure 3B). The iterons for pTZ2162 and pSK41 have repeats of 9 and 8bp respectively. Repeat 3 in PLE has inverted repeats that are longer at 13bp, while those in repeat 4 are only 3bp long. Most characterized RepA_N iteron inverted repeats are at least 5bp long, but obvious inverted repeats are not always discernible (45). To determine if PLE’s RepA is capable of binding to the repetitive sequences in NCR3, we purified RepA and assessed binding to repeat 3 and repeat 4 through an EMSA (Figure 6A). When RepA was titrated into our reactions we observed that the repeat 3 probe, but not the repeat 4 probe was shifted on the gel, confirming that RepA binds the repeat 3 sequence (Figure 6B). Additional genetic analysis showed that the repeat 3 sequence was necessary for PLE replication, but the repeat 4 sequence was not, further supporting our conclusion that repeat 3 serves as the iteron sequence in the PLE ori (Figure 6C).

### Non-replicating PLE alters ICP1 replication dynamics without lowering ICP1 genome copy

Having identified the necessary components of the PLE replicon, we sought to assess the importance of PLE replication for the PLE’s life cycle and anti-phage activity. Following excision and replication, PLE can be transduced to recipient *V. cholerae* cells (12). We hypothesized that PLE replication would be necessary for its transduction, therefore we performed transduction assays, comparing *ΔrepA* PLE complemented with either *repA* or an empty vector control. As expected, PLE transduction was below the limit of detection in *ΔrepA* PLE complemented with an empty vector control (Supplementary Figure S10), and the transduction defect for *ΔrepA* PLE could be complemented by *repA in trans*, restoring PLE transduction to levels near those of wild-type (WT) PLE (3.8 × 10^4^ units/ml, (12)).

**Figure 7.**
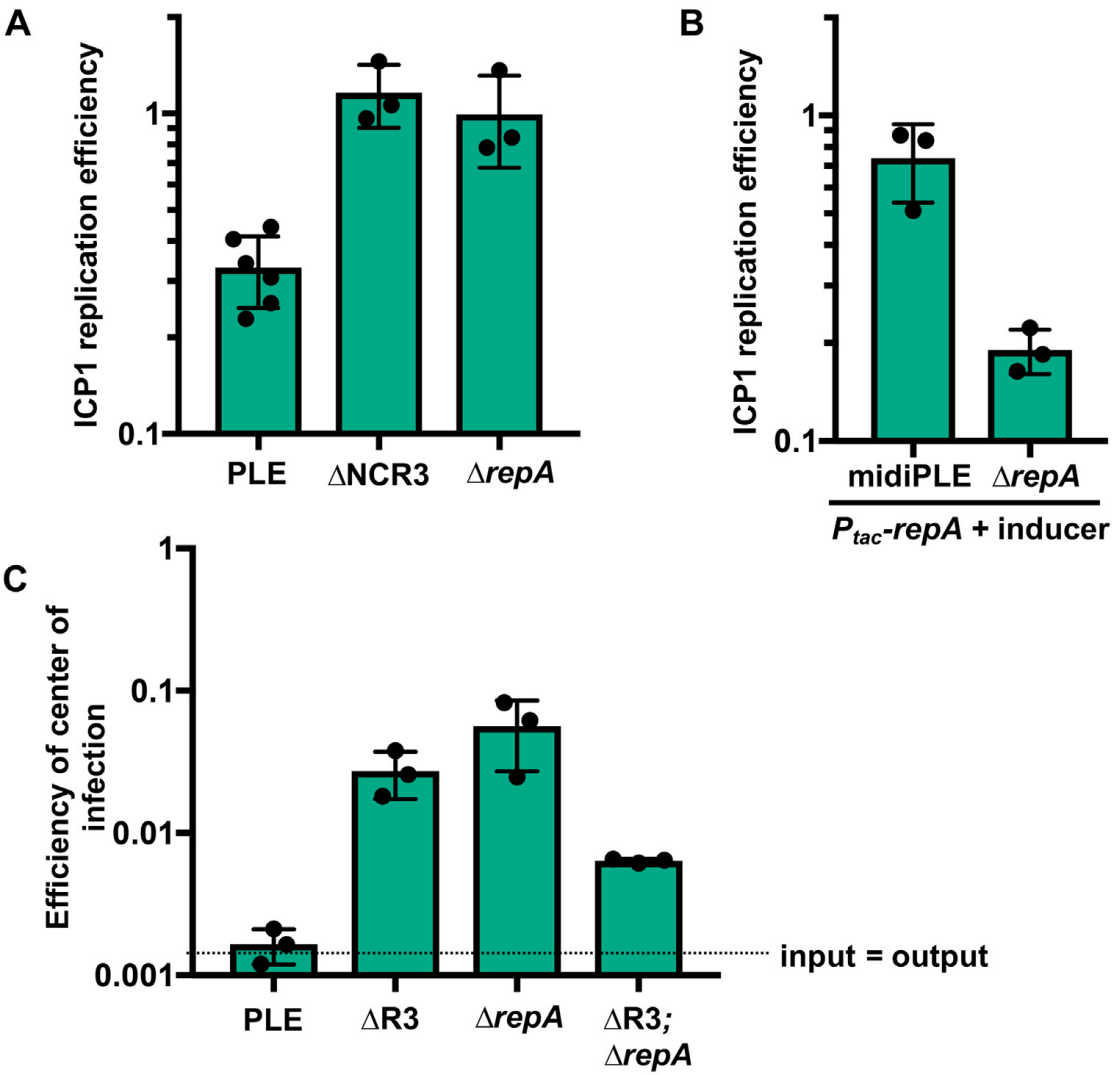
Loss of replication impairs PLE anti-phage activity. (**A**) ICP1 replication in wild-type and mutant PLE(+) strains as assessed by qPCR. Replication efficiency is relative to ICP1 infection of PLE(−) *V. cholerae* 20 min post-infection. (**B**) Replication of ICP1 as assessed by qPCR in RepA complemented midiPLE and *ΔrepA* PLE infection relative to an un-complemented midiPLE control. (**C**) Efficiency of center of infection (EOCI) for ICP1 on wild-type PLE and non-replicating PLE mutant hosts. ECOI is relative to a PLE(−) permissive control strain. The dashed line indicates the threshold at which the number of output phage is equal to the number of input phage. Above the dashed line output has a larger value, below the dashed line input has a larger value.

The finding that high PLE copy is needed to facilitate PLE transduction is intuitive, but under these laboratory conditions PLE produces fewer than one transducing unit per infected cell, despite PLE’s robust replication (12). This lead us to question if PLE replication contributes to PLE’s anti-phage activity. ICP1 replication is reduced in a PLE(+) infection (Figure 2). A potential mechanism of PLE impairment of ICP1 replication could be through the consumption of replication resources. Robust PLE replication might exhaust dNTP pools, and since PLE only replicates during ICP1 infection, PLE might also competitively restrict ICP1’s access to its own replisome. Therefore, we next tested ICP1 replication in non-replicating PLE strains using qPCR, and observed that ICP1 replication was restored to the levels seen in PLE(−) infection conditions (Figure 7A). This restoration led us to question if midiPLE replication could impair ICP1 replication simply by using up replication resources. However, during ectopic expression of *repA*, midiPLE did not impair ICP1 replication, while *ΔrepA* PLE did (Figure 7B). Consistent with this result, we did not observe any defect in ICP1 plaque formation on *V. cholerae* harboring a replicating midiPLE (Supplementary Figure S11). This suggests that PLE replication reduces ICP1 copy through an independent mechanism, which may be dependent on PLE gene dosage increase, or by reaching a level of replication not achievable with the midiPLE.

The roughly 4-fold decrease in ICP1 replication that occurs in PLE(+) cultures would not likely be sufficient for the complete restriction of ICP1 that is observed (12), but is likely to be a contributing mechanism. To investigate this, we performed ICP1 plaque assays on non-replicating PLE mutant hosts. The PLE *ΔrepA* and *Δori* mutants still blocked plaque formation (data not shown), however, the mutants were more susceptible to ICP1 than wild-type PLE as some small plaques were visible when high phage concentrations were added. The small size of these plaques made quantification difficult and less reproducible than desired. Therefore, we quantified ICP1’s efficiency of center of infection (EOCI) on these non-replicating PLE mutants (Figure 7C). Consistent with previous observations (12) virtually no phage were produced from wild-type PLE(+) *V. cholerae*. We did, however, observe an intermediate EOCI on the non-replicating PLE strains. Unexpectedly, a double knockout of both the iteron sequence and *repA* permitted less phage production than each individual knockout. This is peculiar, but could make sense if PLE replication has downstream regulatory effects on PLE activity. Since the iterons and RepA are interacting partners, removing both may allow PLE to bypass any regulatory activities either may have rather than becoming arrested at failed replication initiation. These results illustrate the difficulty of teasing apart direct and downstream effects of PLE replication. Nevertheless, each of our non-replicating PLE mutants had some restoration of phage production, demonstrating that although PLE replication is not necessary for PLE mediated anti-phage activity, PLE replication bolsters or acts synergistically with other PLE-encoded anti-phage activities.

**Figure 8.**
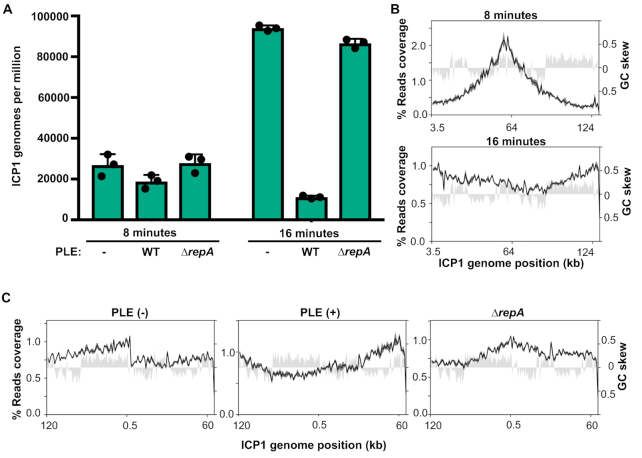
Non-replicating PLE still alters ICP1’s replication profile. (**A**) Genomes per million of ICP1 in PLE(−), PLE(+), and PLE *ΔrepA* cultures at 8 and 16 min post-infection. Values shown are the means of three biological replicates. (**B**) Percent reads coverage profile of ICP1’s genome in *ΔrepA* PLE infection at 8 min (top) and 16 min (bottom) post-infection. (**C**) Percent reads coverage profile of ICP1’s genome in PLE (−), PLE (+), and *ΔrepA* PLE hosts at 16 min post infection. The ICP1 genome has been rotated so that it is centered around the putative rolling circle replication origin. For each reads profile plot in (B) and (C), the -average percent reads coverage across the genome for three biological replicates is shown as a black line, while standard deviation appears as dark gray shading around the line. The GC skew (right axis) is plotted as light gray shading.

We observed that PLE replication decreases the level of ICP1 replication and coverage profiles suggested that PLE inhibits ICP1’s transition to rolling circle replication (Figure 2C). Since qPCR experiments indicated that the level of ICP1 replication was restored when PLE replication was abolished, we last wanted to determine if ICP1’s change in replication mode was also restored when PLE replication was abolished. Therefore we performed deep sequencing of total DNA during an ICP1 infection time course in PLE *ΔrepA* and quantified and mapped coverage from 8 and 16 min post-infection samples. As expected and consistent with the qPCR results, the relative abundance and GPM of ICP1 did not differ from what we saw for the PLE(−) infection conditions (Figure 8A, Supplementary Table S4). As seen before, the coverage profile of ICP1 at 8 min post-infection shows that ICP1 uses a bidirectional mode of replication at that time point. Interestingly, while loss of PLE replication restored ICP1 copy, abundance across the ICP1 genome matched neither the PLE(−) nor wild-type PLE(+) culture conditions at 16 min post-infection (Figure 8B). The highest abundance of reads was shifted near to the end of ICP1’s annotated genome, as in the PLE(−) infection, but the gradual decrease in reads from this point was bidirectional rather than unidirectional (Figure 8C). This reveals that PLE has some capacity to act on ICP1 replication even when the PLE is not replicating, and suggests that PLE may prevent linearization of ICP1’s genome.

## DISCUSSION

Here, we have identified the key constituents of PLE replication and evaluated their importance for ICP1 restriction. Since PLE’s genetic material outnumbers ICP1’s by 16 min post-infection, it is easy to imagine that reduced ICP1 copy, coupled with the presence of a highly abundant competing genome could severely hamper ICP1 packaging. Still, neither a decrease in ICP1 copies, nor a high abundance of PLE copies is necessary for PLE’s anti-phage activity, indicating that PLE has other mechanisms for restricting ICP1. Our results indicate that one of these mechanisms may still be centered around ICP1’s replication even if it does not decrease the overall ICP1 copy. The coverage profiles of ICP1 suggests that it undergoes a transition in replication mode from bidirectional replication to rolling circle replication, and that PLE impedes this transition even when PLE is unable to replicate (Supplementary Figure S12). A number of well characterized phages are known to transition from bidirectional theta to rolling circle replication over the course of infection (26). This transition linearizes and concatenates the phage genome, and concatemeric DNA serves as the packaging substrate for most tailed phages (49). If PLE *ΔrepA* still prevents ICP1 from replicating via a rolling circle mechanism, this could severely impair ICP1’s ability to package its genome. PhageTerm analysis suggests that the ICP1 pac site is proximal to where we predict the rolling circle replication origin, potentially linking rolling circle replication and genome packaging. It is conceivable that the blunt terminus generated by the first round of rolling circle replication could act as a recognition site for the ICP1 terminase, which would then package the concatemeric genome in a headful fashion. Additionally, if a loss of genome linearization is not sufficient to prevent ICP1 particle production, it could act synergistically with other anti-packaging mechanisms such as the capsid hijacking observed in SaPIs and P4.

The precise relationship between ICP1’s and the midiPLE’s (and by extension PLE’s) DNA replication remains unclear. Specifically, it is unclear if replication of the midiPLE does not interfere with ICP1’s replication because the midiPLE does not replicate to the same level as PLE, or if the midiPLE is unable to reach as high of a copy level because ICP1 replication is unperturbed. Further work will be needed to identify factors that act on ICP1 replication without impacting PLE copy.

PLE’s lack of autonomous replication is a striking contrast to previously characterized bacteriophage satellites. PLE’s reliance on ICP1 for replication and not just activation of gene expression indicates that PLE parasitizes ICP1-encoded gene products for replication. Recent work further demonstrates that PLE parasitizes ICP1 proteins for replication as it was found that PLE replication cannot proceed during infection by ICP1 mutants lacking an SF1B-type helicase (50). ICP1 isolates encode one of two SF1B-type helicases (*helA* or *helB*) in syntenic loci. Despite sharing only 24% amino acid identity, each of these helicases can be exploited for PLE replication during infection. Surprisingly, Δ*helA* and Δ*helB* phage can drive PLE replication when complemented by Dda, an even more distantly related SF1B-type helicase encoded by phage T4. This is especially remarkable given that ICP1’s core replisome does not resemble those of T4-like phage (24,26,51). The sequence diversity of these SF1B-type helicases, and the unrelatedness of their cognate phages’ replisomes suggests that it may be their enzymatic activity, rather than a protein binding affinity, that makes the helicases necessary for PLE replication.

Questions remain for understanding PLE replication: specifically, what replication machinery PLE is recruiting to the ori and how that recruitment occurs. Replication proteins for SaPIs and P4 possess primase and helicase activity (21–23) but PLE RepA is not predicted to possess either. How RepA_N proteins initiate replication remains unknown, and given that the C-terminal domain of these proteins are genera specific, it is likely that RepA_N proteins in different bacteria will recruit different machinery and may initiate replication through distinct mechanisms. PLE’s need for an SF1B-type helicase for replication (50) offers some potential clues into how RepA initiates PLE replication. Though the physiological role of Dda in T4 as well as the SF1B-type helicases in ICP1 remains elusive, Dda has been implicated in loading one of T4’s origins of replication, and may be involved in modulation of recombination (26,52). Recombination is coupled to replication initiation during processes such as the restarting of stalled replication forks and the recombination dependent replication carried out by T4 phages (26,52). Rather than recruit ICP1 replication machinery through direct interactions, PLE RepA might be restructuring the PLE origin so that it resembles damaged replication intermediates. ICP1’s SF1B-type helicase may then process the origin so that the ICP1 replisome can load onto that site. Using DNA repair processes as a backdoor for replisome loading could explain how PLE is able to use diverse SF1B-type helicases to replicate. Future work will be needed to explore this possibility.

It is surprising that PLE encodes a RepA_N family initiator given their rarity among Gram-negative bacteria. Of the 742 RepA_N family proteins annotated in the Pfam database, 723 belong either to Firmicutes species or bacteriophage that infect them (Pfam: Family:RepA_N (PF06970)). Only two RepA_N family sequences have been previously identified in Proteobacteria, both of them in the group Burkholderiales. Interestingly, RepA is not the only PLE-encoded gene that belongs to a family that is underrepresented in Gram-negative bacteria. The PLE integrase responsible for excision and integration into the host chromosome is a large serine recombinase (27) another protein type rarely found in Gram-negative bacteria (53). Though PLEs lack any detectable homology to other known satellites, the presence of a large serine recombinase and RepA_N initiator in PLEs raises the possibility of recent inter-phyla gene transfer or deep evolutionary roots for PLEs.

Previously, it was noted that the chromosomally encoded RepA_N family proteins are linked to tyrosine and serine recombinases (45). The authors speculated that these genes were located on conjugative transposons and that the RepA_N had acquired new activities to facilitate transfer, since conjugative transposons, unlike plasmids or phages, do not need to replicate independently of the chromosome. An equally plausible explanation is that these recombinases and RepA_N genes are encoded by cryptic bacteriophage satellites. Supporting this possibility, a *Clostridium difficile* conjugative transposon encoding both a serine recombinase and a RepA_N initiator, as well as erythromycin resistance, was found to be transduced by a phage at a higher frequency of transfer than could be achieved by filter mating (54). This suggests that the boundary between viral satellite and conjugative element may not always be well defined, and individual elements may have some flexibility in their routes of mobilization. Since satellites typically do not encode their own structural genes, there is little to distinguish them from transposons or conjugative elements when one is making sequence based predictions. We anticipate that bacteriophage satellites will be found to be far more common than currently appreciated. Characterization of the PLE offers a window into these fascinating entities that shape the lives of their bacterial, and viral, hosts.

## DATA AVAILABILITY

The sequencing data from phage infected cells generated in this study have been deposited in the Sequence Read Archive database under BioProject accession code PRJNA577694.

## Supplementary Material

gkz1005_Supplemental_Files
