# Supplementary material for: Genome replication dynamics of a bacteriophage and its satellite reveal strategies for parasitism and viral restriction
Source: Nucleic Acids Res. 2019 Oct 31;48(1):249–63. doi: 10.1093/nar/gkz1005 (PMC7145576; doi:10.1093/nar/gkz1005)
Supplement: gkz1005_Supplemental_Files [file gkz1005_supplemental_files.zip › Barth Supp tables revised.docx]

**Supplementary Table S1: Strains used in this study**

| Strain | Description* | Source |
| --- | --- | --- |
| KDS6 | *V. cholerae* O1, El Tor biotype | Lab collection |
| KDS36 | *V. cholerae* E7946 containing PLE1 | (1) |
| KDS153 | *V. cholerae* E7946 PLE1 ΔORF7::frt, KanR | This study |
| KDS154 | *V. cholerae* E7946 PLE1 ΔORF8::frt, KanR | This study |
| KDS155 | *V. cholerae* E7946 PLE1 ΔORF9::frt, KanR | This study |
| KDS156 | *V. cholerae* E7946 PLE1 ΔORF10::frt, KanR | This study |
| KDS157 | *V. cholerae* E7946 PLE1 *ΔrepA*::frt, KanR | This study |
| KDS158 | *V. cholerae* E7946 PLE1 ΔORF12::frt, KanR | This study |
| KDS159 | *V. cholerae* E7946 PLE1 ΔORF12.1::frt, KanR | This study |
| KDS160 | *V. cholerae* E7946 PLE1 ΔORF13::frt, KanR | This study |
| KDS161 | *V. cholerae* E7946 PLE1 ΔORF14::frt, KanR | This study |
| KDS181 | *V. cholerae* E7946 PLE1 *Δint*::Spec-frt | (2) |
| KDS182 | *V. cholerae* E7946 PLE1 ΔORFs2-5::Spec-frt | (2) |
| KDS183 | *V. cholerae* E7946 PLE1 ΔORFs7-14::Spec-frt | (2) |
| KDS184 | *V. cholerae* E7946 PLE1 ΔORFs15-20::Spec-frt | (2) |
| KDS185 | *V. cholerae* E7946 PLE1 ΔORFs21-23::Spec-frt | (2) |
| KDS228 | *V. cholerae* E7946 *ΔlacZ*::SpecR | (1) |
| KDS229 | *V. cholerae* E7946 PLE1 *ΔrepA*::frt, *ΔlacZ*::P*_tac_-repA*, KanR, SpecR (RepA chromosomal expression construct in PLE *ΔrepA*) | This study |
| KDS230 | *V. cholerae* E7946 PLE1 *ΔrepA*::frt, *ΔlacZ*:: P*_tac_* *EV*, KanR, SpecR (Empty chromosomal expression construct in PLE *ΔrepA* ) | This study |
| KDS231 | *V. cholerae* E7946 PLE1 *ΔrepA*::frt, P*_tac_-repA*, KanR, CmR (Plasmid RepA expression construct) | This study |
| KDS232 | *V. cholerae* E7946 midiPLE, P*_tac_-repA*, KanR, SpecR (RepA plasmid expression construct in strain with midiPLE) | This study |
| KDS233 | *V. cholerae* E7946 PLE1 *ΔrepA*::frt, P*_tac_* *EV*, KanR, CmR (Empty plasmid expression construct in PLE *ΔrepA* ) | This study |
| KDS234 | *V. cholerae* E7946 midiPLE, P*_tac_* *EV*, KanR, CmR (Empty plasmid expression construct in strain with midiPLE) | This study |
| KDS235 | *V. cholerae* E7946 PLE1 ΔNCR1, KanR | This study |
| KDS236 | *V. cholerae* E7946 PLE1 ΔNCR2::frt, KanR | This study |
| KDS237 | *V. cholerae* E7946 PLE1 ΔNCR3::frt, KanR | This study |
| KDS238 | *E. coli* BL21 pE-SUMO-RepA. Vector to express 6xHisSumo-fusion protein, fused to N-terminus of RepA | This study |
| KDS263 | *V. cholerae* E7946 PLE1 ∆repeat 3::frt | This study |
| KDS264 | *V. cholerae* E7946 PLE1 ∆repeat 4::frt | This study |
| KDS265 | *V. cholerae* E7946 PLE1 ∆repeat 3::frt, *ΔrepA*::frt, KanR |  |
| ICP1 | ICP1_2006_E ΔCRISPR ΔCas2-3 | (2) |

* KanR = Kanamycin resistance cassette, SpecR = Spectinomycin resistance cassette.

**Supplementary Table S2: Primers used in this study**

| Primer | Sequence | Purpose | Source |
| --- | --- | --- | --- |
| zac14 | AGGGTTTGAGTGCGATTACG | qPCR PLE | (1) |
| zac15 | TGAGGTTTTACCACCTTTTGC | qPCR PLE | (1) |
| zac68 | CTGAATCGCCCTACCCGTAC | qPCR ICP1 | (1) |
| zac69 | GTGAACCAACCTTTGTCGCC | qPCR ICP1 | (1) |
| zac109 | CGCCAAACCAACAAGACAGG | qPCR PLE ∆NCR | This study |
| zac110 | CCCCAAGATCAACCACCTCC | qPCR PLE ∆NCR | This study |
| zac205 | TTTATATGGATGATTTTCAACCCTATAAA | F sequence R3 probe | This study |
| zac206 | TTTATAGGGTTGAAAATCATCCATATAAA | R sequence R3 probe | This study |
| zac207 | AAGGTAGACACCTTATGGTAGTTC | F primer R4 probe | This study |
| zac208 | GAACTACCATAAGGTGTCTACCTT | R primer R4 probe | This study |

**Supplementary Table S3: Proportional reads abundance for ICP1, PLE, and the *V. cholerae* chromosomes relative to total sample reads over time course of ICP1 infection**

| **Percent reads per element of total reads per condition** | | | | | | | |
| --- | --- | --- | --- | --- | --- | --- | --- |
|  | PLE(-) infection | | | PLE(+) infection | | | |
| Min^a^ | VC I^b^ | VC II | ICP1 | VC I | VC II | ICP1 | PLE |
| 4 | 79 + 0. 27^c^ | 20 + 0.098 | 1.1 + 0.17 | 79 + 0.26 | 20 + 0.15 | 1.1 + 0.26 | 0. 32 + 8.6E-04 |
| 8 | 78 + 6.0 | 20 + 0.097 | 2.2 + 0.097 | 77 + 0.57 | 19 + 0.14 | 2.5 + 0.61 | 0.67 + 0.12 |
| 12 | 61 + 5.9 | 15 + 1.6 | 24 + 7.4 | 65 + 0.71 | 17 + 0.17 | 14 + 0.71 | 4.5 + 0.29 |
| 16 | 41 + 4.8 | 9.9 + 1.2 | 49 + 6.0 | 51 + 1.3 | 14 + 0.35 | 17 + 0.69 | 19 + 1.4 |

^a^ time in minutes post-infection

^b^ VC I is the *V. cholerae* large chromosome, VCI II is the *V. cholerae* small chromosome

^c^ Values are the mean and standard deviation of three biological replicates

**Supplementary Table S4: Proportional reads abundance for ICP1, PLE, and the *V. cholerae* chromosomes relative to total sample reads over time course of ICP1 infection in PLE *ΔrepA***

| **Percent reads per element of total reads per condition** | | | | |
| --- | --- | --- | --- | --- |
| Min^a^ | VC I | VC II | ICP1 | PLE |
| 8 | 77 + 0.56 | 19 + 0.12 | 30 + 0.62 | 0.31 + 0.013 |
| 16 | 44 + 3.1 | 11 + 0.8 | 45 + 0.39 | 0.24 + 8.5E -03 |

^a^ time in minutes post-infection

^b^ VC I is the *V. cholerae* large chromosome, VCI II is the *V. cholerae* small chromosome

^c^ Values are the mean and standard deviation of three biological replicates

**References**

1. O’Hara,B.J., Barth,Z.K., McKitterick,A.C. and Seed,K.D. (2017) A highly specific phage defense system is a conserved feature of the *Vibrio cholerae* mobilome. *PLoS Genet.*, **13**, e1006838.

2. McKitterick,A.C. and Seed,K.D. (2018) Anti-phage islands force their target phage to directly mediate island excision and spread. *Nat. Commun.*, **9**, 2348.
