# Supplementary material for: Genome replication dynamics of a bacteriophage and its satellite reveal strategies for parasitism and viral restriction
Source: Nucleic Acids Res. 2019 Oct 31;48(1):249–63. doi: 10.1093/nar/gkz1005 (PMC7145576; doi:10.1093/nar/gkz1005)
Supplement: gkz1005_Supplemental_Files [file gkz1005_supplemental_files.zip › Supplementary figures 1-12.pdf]

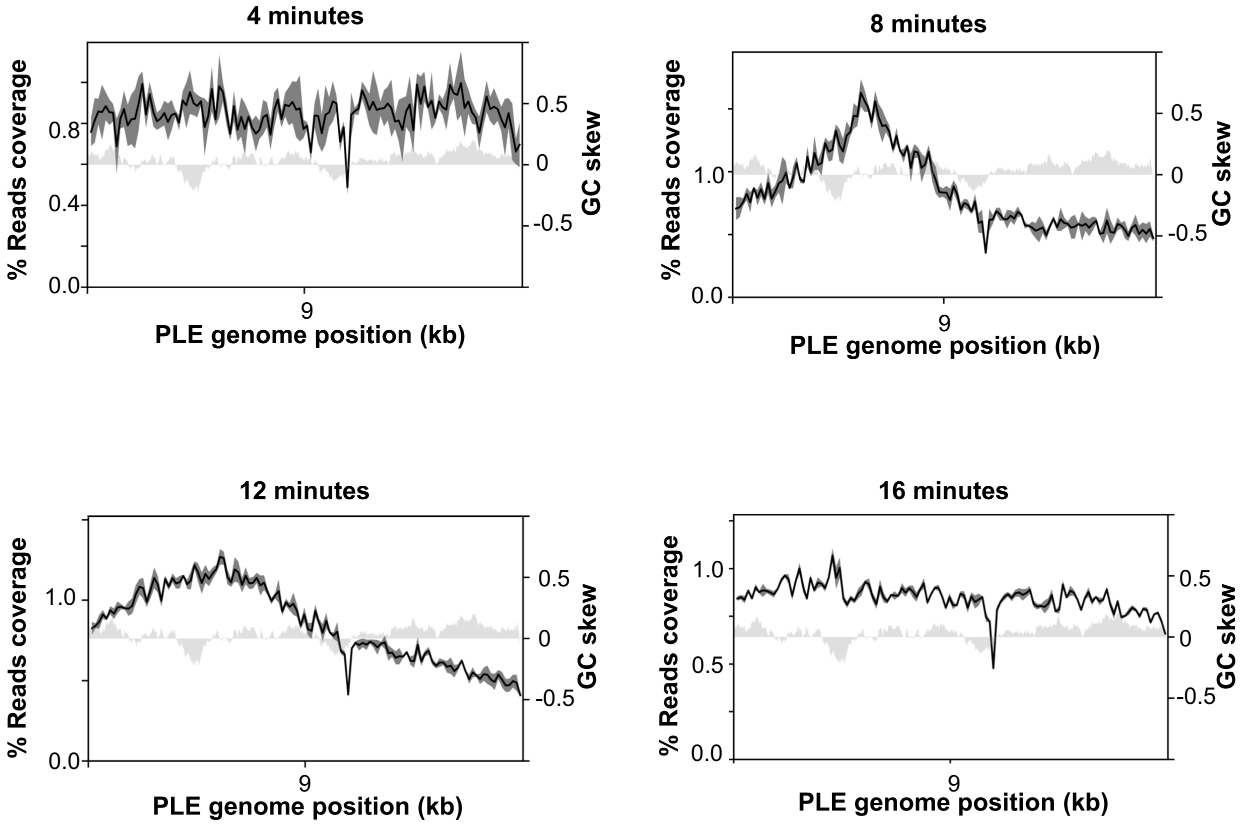

Supplementary Figure 1. Percent reads coverage plots across the PLE genome during ICP1 infection. For each time point, the percent reads coverage across the genome for three biological replicates was determined. The average percent reads coverage is shown as a black line, while standard deviation appears as dark gray shading around the line. The GC skew is plotted on the right axis as light gray shading.

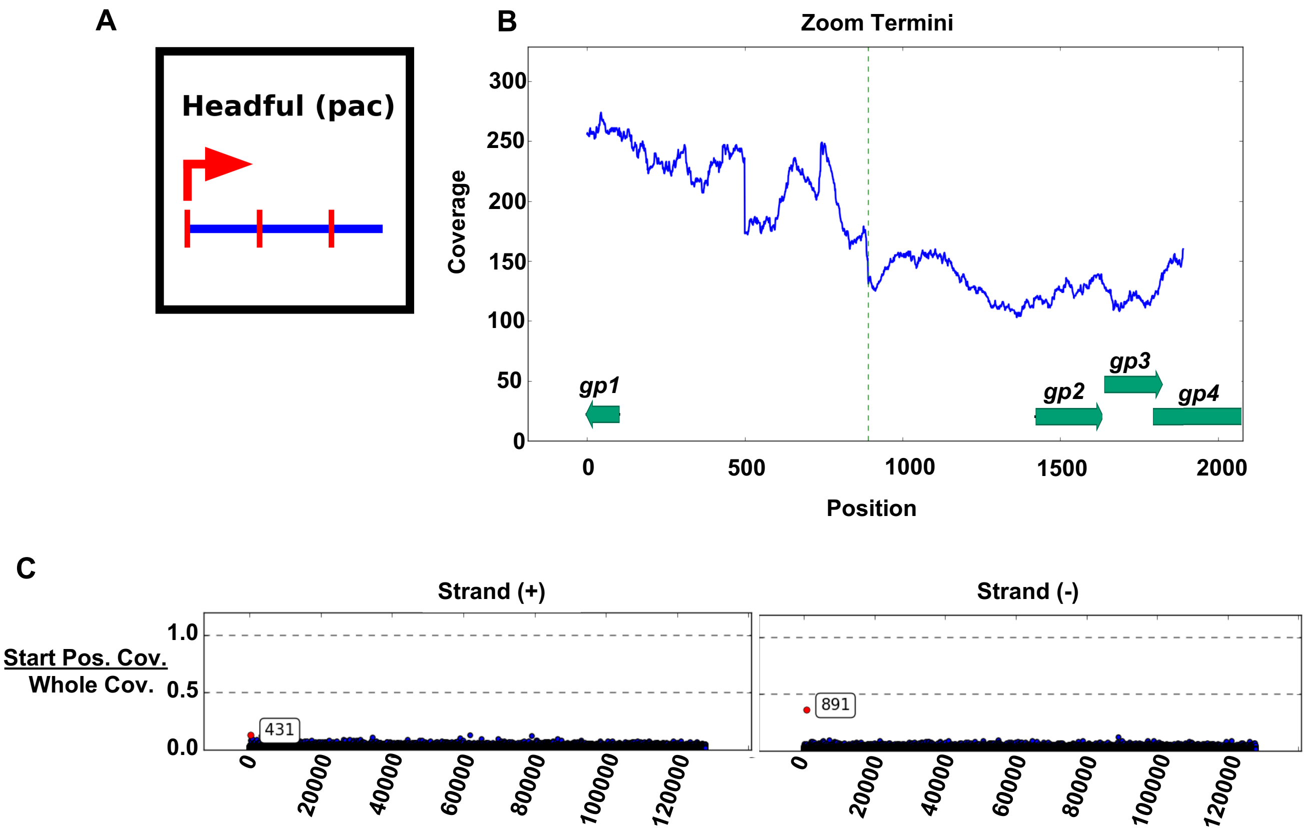

Supplementary Figure 2. ICP1 is predicted to use a headful packaging mechanism dependent on a pac site as determined by PhageTerm analysis. **(A)** PhageTerm schematic showing the predicted packaging mode of ICP1. ICP1 DNA is packaged into capsids using a headful mechanism from a distinct site on the phage genome. **(B)** A zoomed in view of ICP1's packaging terminus with whole genome coverage plotted. **(C)** Plots of reads start position coverage divided by whole coverage along the entire ICP1 genome. The (+) strand (left) and (-) strand (right) are plotted separately.

Repeat 1

```
CAGAACGTCATTTAACGCATCTTAT-CACCACCTTAATA
CAGAACGTCATTTAACGCATTTTACGCACCACCCTAATA
******************** ***  ******* *****
```

Repeat 2

```
ACTTATACGTTAGTATTACTGACGTTAGTATTACCCCCA
ACTTA--CGTTAGTATAACTTACGTTAGTA-TACCCTCA
*****  ********* *** ********* ***** **
```

Repeat 3

```
TTTATAGTTAGTGGGATGATTTTCATACCTATAAA
TTTATAT------GGATGATTTTCAACCCTATAAA
TTTATAG------GTATGATTTTCAGCCCTATAAA
******       * **********  ********
```

Repeat 4

```
AAGTGAGACACCTTATGGTAGTTC
AAGGTAGACACCTTATGGTAGTTC
AAGGTAGACACCTTTTGGTAG---
***  ********* ******
```

Supplementary Figure 3. Repeat sequences found within the PLE noncoding region. Mismatches are shown in light gray, asterisks represent conserved sequence. Repeats 1 and 2 are interspersed with each other across a 528bp region (Figure 3B). The repeat 3 sequences are proximal to each other separated by 5 and 14bp, and the repeat 4 sequences are contiguous.

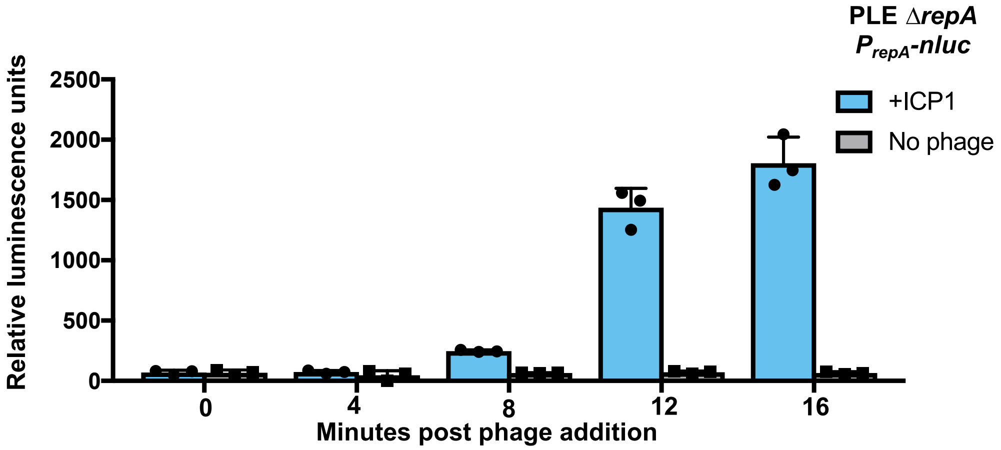

Supplementary Figure 4. Relative luminescence units of a PLE P_{repA}nluc reporter strain, where the repA gene has been replaced by nanoluciferease (nluc). Values shown are the means of three biological replicates.

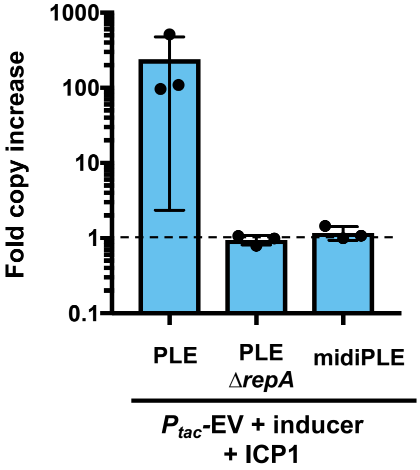

Supplementary Figure 5. Fold copy increase of wild-type PLE, PLE Δ*repA*, and the midiPLE following induction of an empty vector control (EV) 20 minutes post-infection with ICP1 as assessed by qPCR. The dashed line indicates no change in PLE copy.

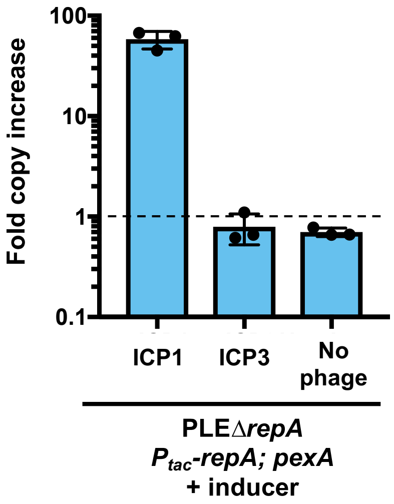

Supplementary Figure 6. Fold copy increase of PLE Δ*repA*, following induction of RepA and PexA expression and addition of ICP1, ICP3, or buffer control 20 minutes post-addition as assessed by qPCR. The dashed line indicates no change in PLE copy.

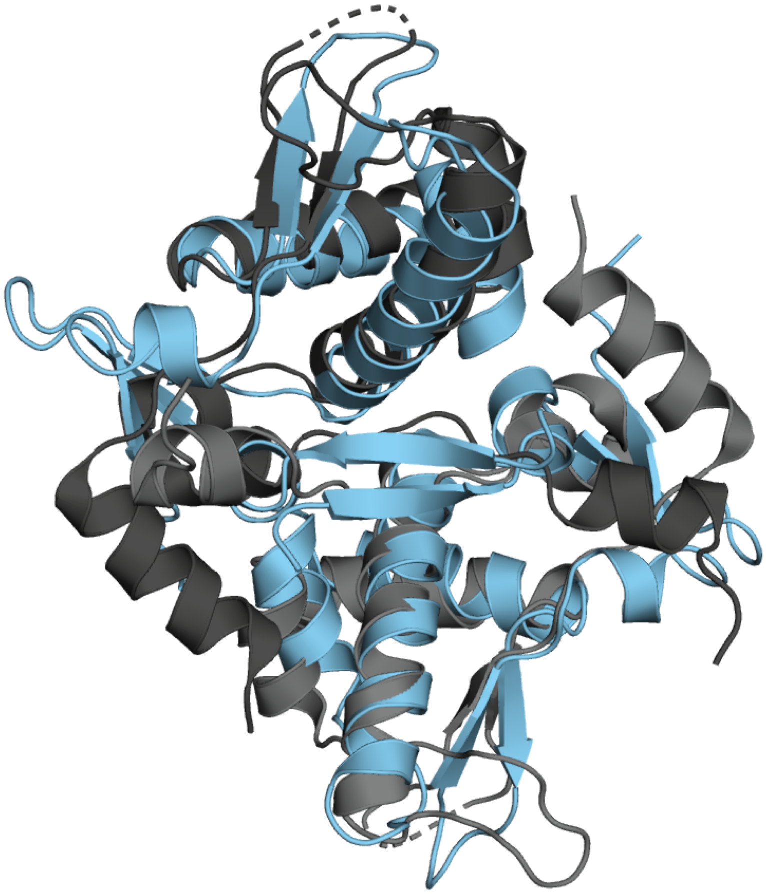

Supplementary Figure 7. Overlay of ribbon diagrams for the crystal structures of the NTD of dimers of PLE RepA (light blue) and pSK41 (dark grey) (RMSD = 4.197942 over 184 residues).

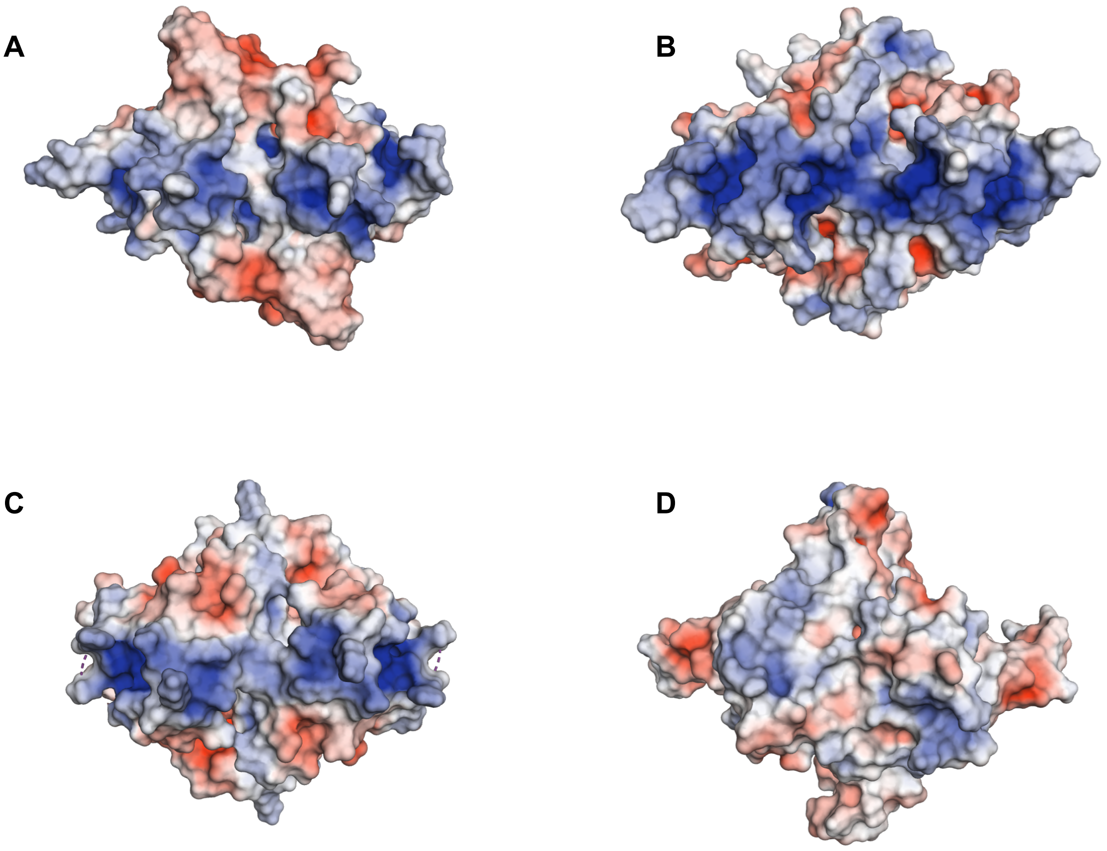

Supplementary Figure 8. Electrostatic profiles for PLE RepA (A), pTZ2162 RepA (B), pSK41 (C), and B. subtilis DnaD (D), N-terminal domain dimers. Positive (blue) and negative (red) charges are indicated on the surface.

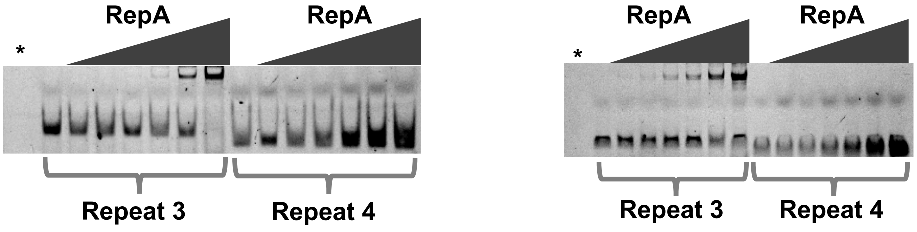

Supplementary Figure 9. Replicates of an electrophoretic mobility shift assay using probes from the PLE noncoding region. RepA binding was tested for probes corresponding to the repeat 3 or repeat 4 sequence from the PLE NCR3. The * denotes a RepA(+) DNA (-) control.

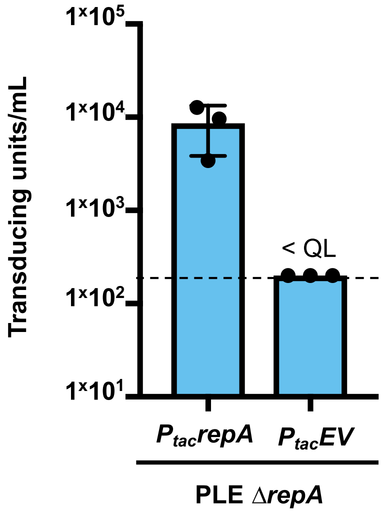

Supplementary Figure 10. PLE replication aids transduction. Transduction units per mL produced from ICP1 infection of Δ*repA* PLE complemented with repA or an empty vector control (EV). Quantification limit (QL) = 200 TU/mL.

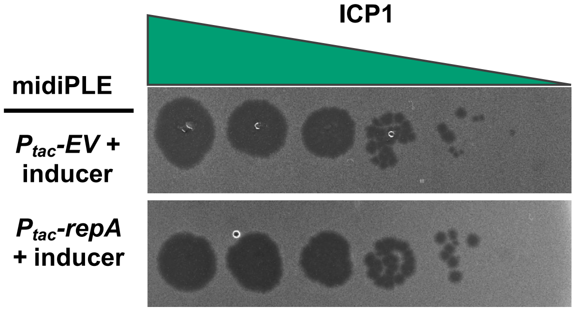

Supplementary Figure 11. Spot assay showing ICP1 susceptibility for *V. cholerae* harboring the midiPLE complemented with an empty vector (EV) or repA. Serial tenfold ICP1 dilutions spotted onto PLE (+) and PLE (-) *V. cholerae* lawns (grey). Zones of cell death are shown in black.

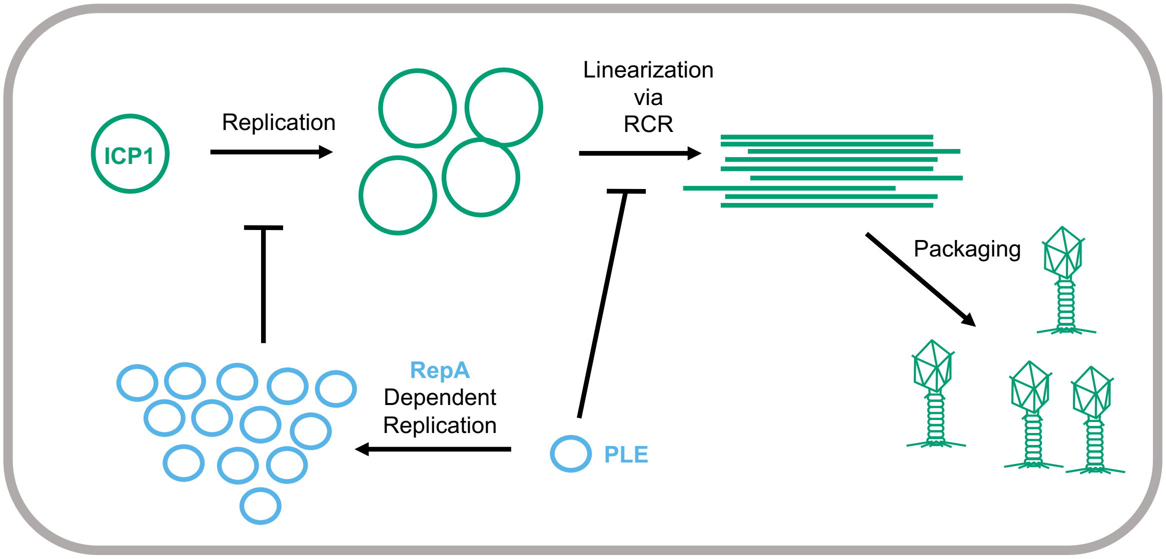

Supplementary Figure 12. Model of PLE interference of ICP1 replication. ICP1 begins replication through a bidirectional theta mechanism before switching to rolling circle replication (RCR). RCR linearizes the ICP1 genome so that it can be packaged into capsids. The PLE is able to block ICP1 linearization without replicating. PLE inhibition of ICP1 copy increase is dependent on PLE replication.
